# How Does Accountability Exacerbate Job Burnout in the Public Sector? Exploratory Research in Production Supervision in China

**DOI:** 10.3390/bs15060747

**Published:** 2025-05-29

**Authors:** Zhiyi Fang, Qilin Zhang

**Affiliations:** School of Political Science & Public Administration, Wuhan University, Wuhan 430072, China; qilinzhang@whu.edu.cn

**Keywords:** felt accountability, role overload, public service motivation, job burnout, moderated mediation model

## Abstract

With the advancement of behavioral public administration, the methodological innovation it introduces offers robust support for investigating the relationship between accountability and individuals’ behavior in the public sector. This paper investigates the link between individuals’ felt accountability and job burnout within China’s industrial production context. We develop and validate a novel theoretical model grounded in cognitive stress theory using survey data collected from public sector safety supervision regulators. The results indicate that the regulators’ felt accountability significantly impacts their job burnout, while threat appraisal played a mediating role in this relationship. Public service motivation negatively moderates the effect, with a more substantial effect observed at higher levels of motivation. This paper contributes to the development of felt accountability theory and promotes interdisciplinary dialogue.

## 1. Introduction

Over the past decade, China’s industrial production supervision accountability system has experienced significant transformations. The “August 12” major fire and explosion accident at Tianjin Port in 2015 marked a pivotal turning point in the development of industrial safety supervision in China. This catastrophic event, which resulted in the tragic loss of 173 lives, catalyzed the revision of the strictest-ever “Work Safety Law” and the introduction of an accountability mechanism emphasizing “shared responsibility between the Party and government, as well as dual responsibility for each post” (Wang et al., 2018). This system design, characterized by strict accountability, has demonstrated a deterrent effect in promoting responsibility fulfillment. However, its derivative effects are increasingly drawing academic attention, as institutional pressure, akin to the Sword of Damocles, may lead to unintended behavioral consequences.

Accountability does not always guarantee proper individual behavior (Ossege, 2012). Some Chinese scholars have observed that the distortion of incentives resulting from an excessive level of accountability has rendered the position of deputies in charge of production safety undesirable, with no individuals willing to assume the role (Lin & Zhao, 2020). Based on in-depth fieldwork, we found that while accountability for production accidents affects administrative leaders, it also leads to the alienation of regulators’ actions in production safety supervision, causing some regulators to exhibit exhaustion and inefficiency, typical characteristics of job burnout. Thus, this paper aims to propose and address a central question: How does the accountability system affect civil servants’ job burnout through micro-psychological mechanisms? What are the specific pathways and boundary conditions of this influence?

As public administration increasingly prioritizes micro-level research, scholars have begun to focus on the concept of felt accountability. It refers to “an explicit or implicit expectation that a person’s decisions or actions will be evaluated by a salient audience, and rewards or sanctions are believed to depend on this expected evaluation” (Hall et al., 2003). Individuals can cognitively process the accountability system and subsequently modify their behavioral patterns and decision-making (Overman, 2021). Felt accountability thus serves as a “bridge” connecting macro-level accountability systems with micro-level individual behavior; research grounded in this concept is better positioned to uncover the transmission mechanisms linking macro institutions with micro behaviors. Job burnout is a characteristic chronic stress syndrome that refers to the long-term reactions individuals experience due to the excessive loss of energy and resources at work (Freudenberger, 1974). Maslach and Jackson (1981) divided it into three dimensions: exhaustion, cynicism, and inefficacy. Job burnout is an important mediator in the behavioral alienation caused by the work environment, leading to consequences such as decreased job satisfaction, lower work performance, and increased willingness to resign (Maslach & Leiter, 2000).

While recent public administration scholarship has increasingly studied felt accountability, critical aspects—particularly its influence on behavioral outcomes—remain limited (Overman, 2021), and the understanding of felt accountability in specific occupations is still vague. Academia is still far from understanding the psychological basis of accountability (Aleksovska et al., 2022). Accountability systems can only be maximized if individuals effectively perceive them (Schillemans et al., 2021); a more profound comprehension of how accountability influences individual behavior is essential for achieving enhanced public governance effectiveness. This article examines the relationship between felt accountability and job burnout from the perspective of behavioral public administration. It is an emerging interdisciplinary field that combines public administration and psychology (Grimmelikhuijsen et al., 2017) and offers a basic methodology for exploring this issue. Given that job burnout reflects an individual’s chronic response to stress, this paper incorporates the cognitive theory of stress, establishes a logical framework of “institutional pressure–threat appraisal–burnout reaction”, and investigates the mediating role of threat appraisal. Additionally, it introduces public service motivation, a key individual-level concept in public administration research, to examine its moderating effect.

The theoretical contributions of this study are reflected in three aspects: first, it transcends the traditional institutionalism paradigm and deconstructs the micro-mechanism of the accountability system from the psychological cognition perspective; second, it enriches the institutional context dimension in job burnout research and elucidates the unique pressure transmission mechanism of administrative accountability; third, by examining the buffering effect of public service motivation, it offers a theoretical foundation for differentiated incentives in institutional optimization. The research findings provide practical insights into refining the institutional design for responsible government construction and mitigating burnout syndrome in grassroots governance.

## 2. Literature Review

### 2.1. Felt Accountability

In public administration research, some scholars have based their study on the original definition (Schillemans et al., 2021), while some scholars have defined felt accountability as an expectation that one may be asked to explain their action to the salient audience(s) with the belief of a consequence based on evaluation (Han & Perry, 2020a). The distinction between the two concepts is reflected in the number of accountability stakeholders. But overall, the pre-existing concept is centered on individuals’ expectations; it encompasses the idea of behaviors that may be subject to sanctions or deemed appropriate within one’s accountability environment (Olsen, 2017), and accountability is conceptualized as a subjective state rather than an objective reality.

Although the perspective highlights the subjective and internal nature of accountability, one’s felt accountability is also dependent on professional role identity (Katz & Kahn, 2015). Existing research has identified archetypes of public sector employees that diverge from the generic accountability relationship observed in psychology (Overman & Schilleman, 2022). Specialized regulators, who adhere to professional norms and apply institutionalized values and procedures, serve as one such example. This group forms the central focus of this paper. Specifically, this paper takes regulators engaged in safety supervision in industrial production as the research subjects. In the public sector, account givers play a role within the broader accountability environment, which is composed of multiple account givers and account holders. Therefore, public sector regulators operate within complex networks of accountability (Page, 2006). Their accountability lies with professional, bureaucratic, political, and social accountability (Goodin et al., 2014), and their actions are evaluated by professional norms, political principles, administrative procedures, and the public. As for the question of “accountability for what?”, given that production supervision accountability is based on post-event outcomes, regulators are held accountable for achieving their specific goal (Schillemans, 2016): minimizing the likelihood of accidents to the greatest extent possible.

### 2.2. Job Burnout

Early analyses of the causes of job burnout mainly focused on internal individual characteristics and external environmental factors (Anderson & Iwanicki, 1984; Schaufeli & Enzmann, 1998). Later, scholars realized that job burnout occurs in a specific work environment, so they tried to analyze the causes of job burnout from the perspective of individual–environment interactions and formed two crucial theories: the Job Demands–Resource Model and the Conservation of Resources (COR) model. The JD-R model holds that any work environment can be characterized by job demands and job resources (Demerouti & Bakker, 2011). Research conducted over the past few decades has revealed that high job demands and low job resources constitute a high-stress work environment, which can lead to chronic job burnout (Bakker & Demerouti, 2017). The COR model holds that resources are the essential unit for understanding stress, and burnout emerges due to persistent threats to available resources (Hobfoll et al., 2018). The loss of resources, or even the anticipation of resource loss, may exacerbate burnout. In essence, job burnout is an individual’s response to stress. Being in a high-pressure situation for a long time will cause an individual to experience job burnout (Maslach et al., 2001). Simultaneously, resource endowment exerts a crucial impact on individual job burnout. In situations where individuals lack resources to manage work demands, the likelihood of intensified job burnout significantly increases.

### 2.3. The Relationship Between Felt Accountability and Job Burnout

As previously discussed, job burnout represents an individual’s reaction to prolonged exposure to work-related stressors. Since individuals must adhere to the role-based behavioral norms expected by account holders, felt accountability is a stressor that can stimulate individuals to engage in different behaviors (Hall et al., 2006; Mackey et al., 2018). This offers fundamental evidence for establishing the relationship between felt accountability and job burnout.

Existing research has shown that an individual’s accountability networks may lack alignment, and conflict and confusion may arise when accountability networks are misaligned (Gelfand et al., 2004). For regulators, a distortion of accountability arises from the misalignment between political and professional accountability. During China’s extensive economic development phase, the professional accountability goals of regulators were established by the administrative accountability system and aligned with the social accountability system. Given the government’s limited focus on industrial production safety supervision during this period, the influence of political accountability was relatively constrained. However, as the state places growing emphasis on production safety, production accidents, particularly those involving casualties, are likely to exert a substantial negative influence on the promotion prospects of local political leaders (Nie et al., 2013). Consequently, the political accountability mechanism has been markedly reinforced. To safeguard their interests to the greatest extent, local political leaders frequently insist on the complete prevention of safety accidents, a standard that surpasses the requirements of other accountability systems.

Industrial production is characterized by high complexity and unpredictability. The foundation of work safety in China remains to be strengthened. The systems for identifying potential safety hazards and implementing full-chain rectification responsibilities require further development. Moreover, enterprises have yet to fully assume their safety obligations. Therefore, regulators face significant challenges in fully achieving the regulatory goal of mitigating accident risks solely through individual efforts. According to the JD-R and COR models, they are placed in a context where their limited individual resources make it challenging to effectively address high job demands, potentially exacerbating their job burnout. When faced with unattainable policy goals, individuals may engage in passive responsibility-avoidance behaviors to evade punishment for failure (Hood, 2013). However, unlike political officials, regulators lack the autonomy to engage in evasive behavior (Bovens et al., 2008). Therefore, they are compelled to confront situations beyond their management capabilities. In such contexts, regulators may first experience significant emotional exhaustion (Hall et al., 2006). Moreover, political accountability constrains their autonomy and power to perform their work by professional procedures, potentially leading to cynicism and inefficiency (Maslach & Leiter, 2016).

**H1:**  *Felt accountability of regulators is positively associated with job burnout*.

### 2.4. The Mediating Role of Threat Appraisal

A stressor is a factor that can impede or facilitate the achievement of an individual’s goals due to its dualistic nature (Cavanaugh et al., 2000). Consequently, an individual’s perception of stress is critical in determining their response. The cognitive theory of stress elucidates individuals’ cognitive evaluation process and corresponding response mechanisms when facing stress (Lazarus et al., 1985), and it is widely applied to explore individuals’ differentiated evaluation and response to stressors. It suggests that the interaction between an individual and their environment prompts the stressor assessment, which in turn shapes the individual’s interpretation of these stressors and influences their subsequent differentiated responses (Folkman & Lazarus, 1986). Sufficient resources are crucial in this process (Lazarus & Folkman, 1987). Specifically, when individuals perceive the demands of a situation as opportunities for growth and possess sufficient resources to address them, they are more likely to evaluate the stressor as a challenge and exhibit positive responses. Conversely, if individuals believe that the stressor will exhaust their resources and be difficult to manage effectively, they will appraise it as a threat. As discussed earlier, regulators face a pronounced disparity between work demands and available resources within the current accountability framework. Consequently, they are highly likely to feel accountability as a potential threat. When regulators feel accountability as a threat, they are likely to exhibit a range of negative responses (Lazarus & Folkman, 1987). Existing research has confirmed the mediating role of threat appraisal in the relationship between stress and job burnout (Gomes et al., 2013). Therefore, this study posits that regulators’ threat appraisal of accountability may mediate the relationship between felt accountability and job burnout.

**H2:**  *Threat appraisal plays a mediating role in the relationship between felt accountability and job burnout*.

### 2.5. The Moderating Role of Public Service Motivation

Public service motivation (PSM) is one of the most essential concepts in studying the intrinsic motivation driving public sector employees. This article introduces PSM to enhance the theoretical understanding of internal changes within individuals and develop a more comprehensive cognitive framework. This concept refers to “beliefs, values and attitudes that transcend self-interest and organizational interests, which involve the interests of larger political entities and motivate individuals to take corresponding actions at appropriate times” (Vandenabeele, 2007).

Recent research findings are inconsistent regarding the relationship between public service motivation and job burnout. Some studies suggest that public service motivation is an important internal resource and a positive intrinsic motivation of an individual, potent for alleviating job burnout (Resh et al., 2018). However, some studies have found that individuals with high public service motivation are more prone to job burnout (Noesgaard & Hansen, 2018). As the investigation unfolds, some studies have pointed out that public service motivation has a double-edged sword effect on job burnout (Zhang & Liu, 2023). Overall, the mechanism of public service motivation on job burnout is relatively complex and should be further discussed in specific research contexts.

This paper argues that the key to exploring the relationship between public service motivation and job burnout lies in determining the attributes of public service motivation. Public service motivation exhibits the dual characteristics of individual resources and individual needs (Giauque et al., 2013). Its resource attribute can reduce the individual’s perception of pressure and alleviate job burnout. In contrast, its demand attribute can increase individual expectations and lead to increased individual pressure and increased job burnout. Studies have shown that individuals are more likely to experience psychological retreat and turn to self-protection processes in high-demand situations, using individual resources as coping mechanisms or stress-relief behaviors (Demerouti & Bakker, 2011; Hopstaken et al., 2014). Based on this, this paper posits that, in the context of safety supervision, the need attributes of regulators’ public service motivation will diminish, whereas the resource attributes will be strengthened. As an individual’s internal resource, the latter will play a role in alleviating job burnout, with the two being negatively correlated.

**H3:**  *Public service motivation serves as a negative moderator in the relationship between felt accountability, threat appraisal and job burnout.*

**Figure 1:**
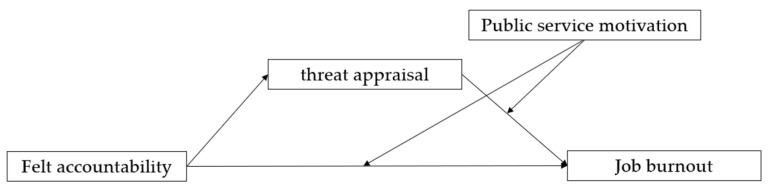
Moderated mediation model.

The variable model presented in this article is illustrated in Figure 1.

## 3. Materials and Methods

### 3.1. Research Sample and Data Collection

This article focuses on civil servants engaged in production supervision as the research subject, employs a cross-sectional study, and collects data via the “Cremado” platform, a professional data collection platform whose data quality has been widely acknowledged by the academic community (Liu et al., 2024). To align the data with the research objectives of this article, we designed a set of questionnaires to categorize the respondents and utilized the platform’s built-in functions to ensure that all respondents were civil servants primarily. Additionally, we included a note in the questionnaire indicating that it served for pre-survey purposes. If respondents met our research criteria, we promptly sent them a formal questionnaire and enhanced participation incentives, encouraging them to review the accompanying instructions carefully.

Civil servants responding to the questionnaire were required to answer whether their work was related to industrial production inspection; those whose work was unrelated were excluded from the study. At the same time, respondents were required to answer some questions that only regulators in industrial production supervision would be familiar with, such as “is the State Mine Safety Supervision Bureau subordinate to the Emergency Management Department at the provincial level?” or “is the core work of the three-year action plan to tackle the root causes of production safety?”. If the respondents answered incorrectly, they were probably not the person of interest and were excluded.

On this basis, we further categorized those who passed the inspection above by industry and position. On the one hand, because our study focused on industrial production supervision, regulators associated with road traffic safety and construction were excluded. The respondents who passed the screening mainly came from the trade, mining, and hazardous chemicals industries. On the other hand, given that participants involved in industrial production supervision held various positions, we excluded those involved in logistical administration who did not participate in actual supervision.

We then compiled a sample pool of respondents who passed all screening tests, issued our formal questionnaire to that group and obtained their informed consent. We distributed 530 questionnaires, with a response rate of 91%, and 482 questionnaires were collected. Since the platform records the IP addresses of respondents, the data show that the respondents cover about half of the regions of China.

### 3.2. Variable Measurement

We employed the translated scale developed by Han and Perry (2020b), which was specifically designed for public sector employees, to measure the felt accountability. This scale categorizes felt accountability into five dimensions: attributability, observability, evaluability, answerability, and consequentiality. It comprises 15 items and uses the Likert seven-point scale.

Threat appraisal was measured using the translated version of the threat appraisal scale developed by Drach-Zahavy and Erez (2002). This scale has been extensively applied in the Chinese management context and has exhibited excellent psychometric properties, indicating high reliability and validity. This section consists of 4 items and employs the Likert seven-point scale.

Regarding the measurement of public service motivation, this article chooses the translated version of the Public Service Motivation Scale developed by Wright et al. (2013), the most widely used scale to measure public service motivation. It divides public service motivation into three dimensions: public interest, compassion, and self-sacrifice, and has good reliability and validity in the Chinese context (Cai & Hu, 2022). It has 5 items and uses the Likert seven-point scale.

Regarding the measurement of job burnout, this paper selected the Job Burnout Scale developed by C. P. Li and Shi (2003), a translated and revised version of the original MBI-GS (Maslach & Jackson, 1981) scale for the Chinese context. The scale divides job burnout into three dimensions: emotional exhaustion, depersonalization, and low achievement motivation. This part has 15 items in total and uses a Likert seven-point scale.

This paper uses age, gender, education level, and years of work experience as control variables to eliminate the possible impact of individual differences on the relationship between variables.

The variable indicators included in the questionnaire are shown in Table 1 below:

## 4. Results

### 4.1. Scale Reliability and Validity Test and Common Method Bias Test

#### 4.1.1. Reliability and Validity Test

Before the hypothesis test, this paper tested the scale’s reliability and validity. The results are shown in Table 2 and Table 3. The Cronbach’s coefficients of all of the variables exceeded 0.7, indicating good reliability. The scale’s component reliability (CR) was greater than 0.7, and the average extracted variance (AVE) was greater than 0.36, confirming convergent validity. A confirmatory factor analysis (CFA) showed that the original model had the best fitting effect, indicating discriminant validity.

#### 4.1.2. Homologous Bias Test

This paper uses two methods to test the data’s homology, with the results shown in Table 3. First, the confirmatory factor analysis shows that the single-factor model has the worst fit. Second, using the ULMC method, adding the common method factor to the original model results in insignificant changes in fit indices (ΔRMR = 0.016, ΔIFI = 0.01, ΔTLI = 0.01, ΔCFI = 0.009, ΔRMSEA = 0.008). These findings suggest that the data do not exhibit serious homology bias.

### 4.2. Descriptive Statistics and Correlation Analysis

The descriptive statistics of each variable are shown in Table 4.

Table 5 shows the correlation analysis between variables. The results show that threat appraisal is positively correlated with the felt accountability (B = 0.778, *p* < 0.001). Job burnout is significantly positively correlated with the felt accountability (B = 0.805, *p* < 0.001) and threat appraisal (B = 0.803, *p* < 0.001), but negatively with public service motivation (B = −0.206, *p* < 0.001). The result aligns with the original hypothesis and supports the subsequent path test.

### 4.3. Main Effect Test and Mediation Effect Test

To avoid multicollinearity interference with the research results, this paper conducts a VIF test for collinearity diagnosis. As shown in Table 6, all variables have VIF values far below 10, indicating no serious multicollinearity issues.

This study employs the PROCESS plug-in in SPSS27 to examine the mediating effect. The tool follows a rigorous stepwise procedure for mediation analysis and is widely used in management research. We selected 5000 samplings with a 95% confidence interval. The results are presented in Table 7. It indicate that the direct predictive effect of the felt accountability on job burnout is significant (c = 0.806 ***), thereby supporting Hypothesis H1. With threat appraisal as a mediator, the direct and indirect effect sizes are 0.463 *** and 0.343 ***, respectively. Since neither the direct nor the indirect effect’s 95% confidence intervals include zero, the mediating effect of threat assessment is confirmed as being significant. Thus, threat appraisal partially mediates the relationship between felt accountability and job burnout, supporting Hypothesis 2.

### 4.4. Moderated Mediation Effect Test

Using Model 15, the moderated mediation effect was examined to evaluate how the mediator’s influence is conditioned by the moderator. As presented in Table 8, the interaction terms of felt accountability and public service motivation (B = −0.084, *p* < 0.05), as well as threat appraisal and public service motivation (B = 0.068, *p* < 0.05), are statistically significant. Consequently, the effects of the felt accountability and threat appraisal on job burnout vary significantly depending on the levels of the moderating variables. Hypothesis 3 is supported.

**Figure 2:**
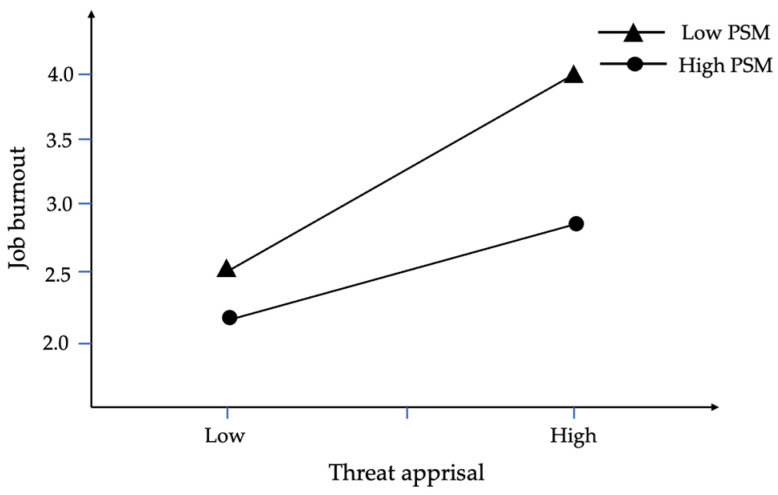
The moderating effect of public service motivation on the influence of threat appraisal on job burnout.

**Figure 3:**
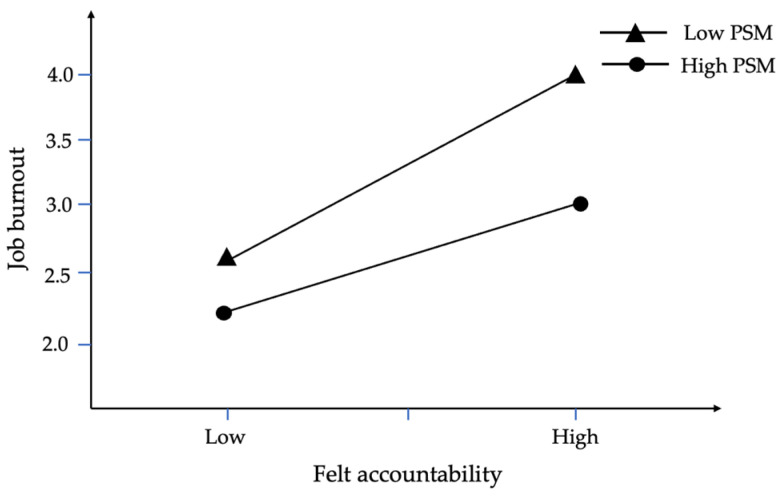
The moderating effect of public service motivation on the influence of felt accountability on job burnout.

To further investigate the moderating role of public service motivation, this study performed a simple slope analysis. The indirect effects (shown in Table 9) reveal that the boot 95% CI does not include zero at low, mean, and high levels, confirming Hypothesis 3. Figure 2 and Figure 3 indicate that individuals with low public service motivation exhibit more pronounced job burnout under the combined influence of felt accountability and threat appraisal, whereas those with high public service motivation can better mitigate the adverse effects associated with stress.

## 5. Discussion and Implications

### 5.1. Main Findings

The significance of exploring the influence of accountability in public administration on individual behavior is becoming increasingly evident (Aleksovska et al., 2022). How-ever, the explanatory power of existing public administration theories is somewhat limited, and research on this issue would benefit from drawing on well-established psycho-logical theories. To address how felt accountability contributes to job burnout, we developed and validated a moderated mediation model grounded in the cognitive theory of stress. The results indicate that the felt accountability, as a stressor, can contribute to job burnout among regulators in the public sector. This finding aligns with conclusions from other research areas, which suggest that felt accountability may lead to job tension and emotional exhaustion (Hochwarter et al., 2005; Hall et al., 2006). Meanwhile, the effect size of 0.806 suggests that felt accountability is a primary contributor to the exacerbation of burnout among regulators. The mediation analysis results reveal that the regulators’ felt accountability prompted them to engage in threat appraisals, intensifying their job burnout. A proportion of 42% of the effect of felt accountability on job burnout is mediated through threat appraisal, suggesting that threat appraisal predominantly mediates this relationship. Concerning the moderating effect, public service motivation moderates the relationship between felt accountability and threat appraisal on job burnout. This suggests that public service motivation moderates the process through which threatening appraisals intensify job burnout, with lower levels of public service motivation exhibiting a weaker moderating effect.

### 5.2. Implications

The theoretical contributions of this paper are extending the research on felt accountability within the field of public administration. Through the development of a model, we confirm the relationship between felt accountability and job burnout, while also investigating individuals’ internal information processing mechanisms.

In business administration, studies investigating the impact of felt accountability on individuals’ work attitudes and behaviors have yielded several significant insights. For instance, empirical research has confirmed that felt accountability positively influences innovative work behaviors (Mero et al., 2014) and enhances job performance (Breaux et al., 2009). Nevertheless, prior studies have also highlighted potential negative consequences, such as increased job strain (Kuo et al., 2022) and heightened feelings of insecurity (Mackey et al., 2018). Thus, investigating further the underlying mechanisms through which felt accountability affects individual behavior represents a critical research agenda. Prior research has highlighted individual autonomy (Hall et al., 2006), while other studies have examined the double-edged nature of felt accountability through the lens of self-determination theory (Y. W. Li et al., 2022). However, the research perspective that focuses on individual initiative somewhat neglects the crucial influence of external conditions on it. This paper incorporates the cognitive theory of stress, emphasizing the process through which individuals perceive external stressors, evaluate them, and subsequently generate behavioral responses, thereby addressing a critical gap in the existing literature. Meanwhile, since this theory only vaguely proposes that individual evaluation outcomes may affect the selection of coping strategies, prior studies have not reached a consistent conclusion regarding the relationship between cognitive evaluation and coping styles. In contrast, this study investigates the relationship between cognitive evaluation and coping styles under conditions where the external environment constrains individuals’ coping options, thereby clarifying the relationship between the two and enhancing its credibility. This contributes new insights into the application of this theory. Furthermore, contemporary scholars in public administration have started to focus on the concept of accountability overload (Rabbi & Sabharwal, 2024), which bears a strong resemblance to the central theme of this study. Consequently, this paper provides a micro-level perspective for exploring this issue.

Furthermore, this research carries significant practical implications. In public management, the formulation and decomposition of policy goals should be grounded in the specific conditions of different regions to enhance goal-setting rationality and avoid scenarios where policy goals become unattainable. Simultaneously, target performance evaluation should exhibit greater flexibility. When performance is suboptimal, rewards and penalties should be tailored based on specific circumstances, moving away from a one-size-fits-all approach.

### 5.3. Research Limitations and Future Directions

This study has limitations. Firstly, this paper focuses on the threat appraisal of felt accountability by regulators in safety supervision. However, considering the substantial variations in the standardization levels of safety production across industries and regions, some regulators with more advantageous objective conditions might see felt accountability as a challenge rather than a threat. This study has not undertaken heterogeneity analyses based on regional or industrial differences, nor incorporated individuals’ evaluations of the challenge dimension of felt accountability and its associated influencing mechanisms into the variable model. Therefore, given that this article primarily focuses on the negative impacts of the misalignment of accountability networks on individuals, it does not delve further into the potential dual-edged nature of felt accountability.

Secondly, the existing literature demonstrates that emotions significantly influence the process of individual information processing (Dewi & Ramantha, 2019). However, this study does not incorporate relevant variables into the variable model. Future research should further extend and refine the existing model by considering these factors.

Thirdly, this study primarily employs cross-sectional research based on survey data. In comparison with qualitative methods such as narrative inquiry or critical incident interviews, this approach may struggle to discern the temporal sequence of variables, which could result in ambiguity regarding causal direction and limit the ability to unpack the black box of mechanisms and elucidate their finer details. Future research should consider utilizing alternative methodologies to further validate this model.

Fourth, this study primarily centers on regulators within the public sector. Overman and Schilleman (2022) highlight that in the public sector, three additional roles—policy makers, CEOs of arm’s-length agencies, and street-level bureaucrats—deviate from the concept of the general account provider as defined in psychology. Consequently, further investigation is warranted to ascertain whether the relationship between felt accountability and job burnout applies to these roles and remains valid across other contexts.

Finally, and most importantly, this study primarily examines the regulators of China’s industrial production. Given the influence of national systems and organizational culture, caution is warranted when considering the generalizability of the findings. However, as research on perceived accountability in China remains in its early stages and lacks comparative studies accounting for cultural differences, further discussion of this issue will be deferred until more theoretical advancements are achieved in this domain.

## 6. Conclusions

In the target approach of public management, accountability serves as a mechanism to ensure the attainment of specific policy objectives. When policy objectives include performance standards that surpass an individual’s capabilities and when individuals face exceptionally high demands on their limited resources, their felt accountability may contribute to job burnout. The results of the empirical research indicate that the effect of felt accountability on job burnout is significant (c = 0.806 ***). In this process, if individuals perceive accountability as a threat and evaluate it accordingly, their job burnout is likely to intensify further. In this study, the direct and indirect mediating effect sizes of threat appraisal are 0.463 *** and 0.343 ***, respectively. Public service motivation can serve as a mitigating factor in reducing job burnout among individuals; the results of the empirical research also support the moderating effect of public service motivation on perceived accountability and the threat assessment on job burnout. From the perspective of behavioral science, the findings of this article essentially present the process of stressors triggering adverse reactions in individuals. Goal orientation, as one of the crucial principles guiding organizational operations, encourages individuals to achieve organizational goals through performance appraisal. However, accomplishing these goals necessitates favorable resource endowments. When organizational objectives exceed an individual’s capabilities, the goals transform into stress, leading to job burnout.

## Figures and Tables

**Table 1:** Latent variables and observed variables of the questionnaire.

Latent Variables	Observed Variables
Attributability (FA1)	FA1-1: What I do is noticed by others in my organization.
	FA1-2: If I make a mistake, I will be caught.
	FA1-3: I am constantly watched to see if I follow my organization’s policies and procedures
Observability (FA2)	FA2-1: Anyone outside my organization can tell whether I’m doing well in my job.
	FA2-2: My errors can be easily spotted outside my organization.
	FA2-3: People outside my organization are interested in my job performance.
Evaluability (FA3)	FA3-1: The outcomes of my work are rigorously evaluated.
	FA3-2: My work efforts are rigorously evaluated.
	FA3-3: I expect to receive frequent feedback from my supervisor.
Answerability (FA4)	FA4-1: I could not easily get away with making a false statement to justify my performance.
	FA4-2: I am always required to follow strict organizational policies or procedures.
	FA4-3: I am not allowed to make excuses to avoid blame in my organization.
Consequentiality (FA5)	FA5-1: If I perform well, I will be rewarded.
	FA5-2: Good effort on my part will ultimately be rewarded.
	FA5-3: If I do my job well, my organization will benefit from it.
Threat appraisal (TA)	TA-1: The task seems like a threat to me.
	TA-2: I’m worried that the task might reveal my weaknesses.
	TA-3: The task seems long and tiresome.
	TA-4: I’m worried that the task might threaten my self-esteem.
Public service motivation (PSM)	PSM-1: It is very important to me to engage in meaningful public service
	PSM-2: Everyday events often remind me that the people in my life are indeed interdependent.
	PSM-3: Contributing to society is more meaningful than personal achievement
	PSM-4: I am ready to make sacrifices for the good of society
	PSM-5: Even if I am ridiculed, I will still strive to fight for the rights of others
Exhaustion (JB-1)	JB1-1: Safe production supervision and law enforcement work makes me physically and mentally exhausted
	JB1-2: I feel exhausted at the end of a day’s work.
	JB1-3: I feel tired when I think about starting a new day at work.
	JB1-4: Working all day is really stressful for me.
	JB1-5: I feel like I’m going to collapse from work.
Cynicism (JB-2)	JB2-1: I am becoming less and less interested in work
	JB2-2: I am not as enthusiastic about my work as before.
	JB2-3: I doubt the meaning of my work
	JB2-4: I care less and less about whether the work I do contributes
Inefficacy (JB-3)	JB3-1: I can effectively solve problems that arise at work
	JB3-2: I feel like I am making a useful contribution to the work
	JB3-3: I think I’m good at my job.
	JB3-4: I feel great when I accomplish something at work.
	JB3-5: I have accomplished a lot of valuable work
	JB3-6: I am confident that I can complete various tasks effectively

**Table 2:** Variable reliability and validity test.

Variable	Cronbach’s α	CR	AVE
Felt accountability	0.881	0.894	0.631
Threat appraisal	0.828	0.832	0.603
Public service motivation	0.912	0.891	0.635
Job burnout	0.887	0.910	0.671

**Table 3:** Confirmatory factor analysis.

	CMIN/DF	RMR	IFI	TLI	CFI	RMSEA
Original model + common factors	2.512	0.041	0.978	0.972	0.977	0.062
Original model	2.487	0.057	0.968	0.962	0.968	0.054
Three factors	2.955	0.068	0.897	0.889	0.897	0.069
Two factors	5.135	0.136	0.788	0.755	0.788	0.093
Single factors	5.538	0.104	0.756	0.733	0.756	0.105

**Table 4:** Descriptive statistics.

	Minimum	Maximum	Mean	Standard Deviation
Felt accountability	1.47	6.47	3.715	0.949
Threat appraisal	1.00	6.75	3.808	1.030
Public service motivation	1.00	6.60	4.573	1.237
Job burnout	1.17	6.46	2.962	0.957

**Table 5:** Correlation analysis.

Variable	Gender	Education	Age	Years of Service	FA	TA	PSM	JB
Gender	1							
Education	−0.050	1						
Age	0.084	−0.106 *	1					
Years of service	0.090	−0.066	0.671 **	1				
FA	−0.015 *	0.159 **	−0.052	−0.091	1			
TA	−0.063	0.116 **	−0.102 **	−0.144 **	0.778 **	1		
PSM	−0.021	−0.085	0.097	0.039	0.186 **	0.169 **	1	
JB	−0.079	0.148 **	−0.109 **	−0.113 **	0.805 **	0.803 **	−0.206 **	1

Note: * represents *p* < 0.01, ** represents *p* < 0.05; double-tailed inspection.

**Table 6:** Collinearity diagnostics.

Variable	FA	TA	PSM	Gender	Age	Education	Years of Serivce
VIF	2.603	2.589	1.064	1.017	1.051	1.848	1.840

**Table 7:** The results of the mediation effect test.

Effect Type	Effect	BootSE	BootLLCI	BootULCI
Total Effect	0.8061	0.0298	0.7475	0.8648
Direct effect	0.4631	0.0417	0.3811	0.5451
Indirect effect	0.3430	0.0355	0.2766	0.4139

Note: LLCI refers to the lower limit of the 95% range of the estimated value, and ULCI refers to the upper limit of the 95% range of the estimated value.

**Table 8:** The test results of the moderated mediation model.

Variable	Threat Apprisal			Job Burnout		
	β	*t*	95%CI	β	*t*	95%CI
Gender	−0.125	−1.565	[−0.282, 0.321]	−0.959	−0.132	[−0.175, −0.016]
Education	−0.133	−0.202	[−0.143, 0.116]	−0.026	0.083	[−0.091, 0.039]
Age	−0.021	−0.330	[−1.145, 0.103]	−0.027	−0.092	[−0.089, 0.035]
Years of service	−0.075	−1.331	[−0.185, 0.036]	0.032	0.040	[−0.023, 0.088]
FA	0.785	21.165 ***	[0.712, 0.858]	0.476	17.223 ***	[0.421, 0.529]
TA				0.432	16.934 ***	[0.381, 0.482]
PSM				−0.282	−18.432 ***	[−0.321, −0.251]
FA*PSM				−0.084	−2.987 ***	[−0.171, −0.070]
TA*PSM				−0.068	−2.582 ***	[−0.132, −0.043]
R^2^	0.542			0.878		
ΔR^2^	0.137			0.115		
F	95.621 ***			286.453 ***		

Note: *** represents *p* < 0.05.

**Table 9:** The results of the conditional indirect effect.

PSM	Effect	BootSE	BootLLCI	BootULCI
Low (−1SD)	0.3839	0.0365	0.3182	0.4628
Mean	0.3390	0.0272	0.2893	0.3951
High (+1SD)	0.2940	0.0328	0.2301	0.3608

## Data Availability

The data presented in this study are available on request from the corresponding author due to privacy and ethical constraints.
